# Evaluation of the Anticancer Properties of Lamellar Alkaloid Drivatives Extracted from the Tunicate *Didemnum abradatum* (Moucha Island Sea, Djibouti): Pharmacological and Computational Approach

**DOI:** 10.3390/molecules30163338

**Published:** 2025-08-11

**Authors:** Fatouma Mohamed Abdoul-Latif, Ibrahim Houmed Aboubaker, Houda Mohamed, Ayoub Ainane, Mouhcine Chakrouni, Ali Merito Ali, Pannaga Pavan Jutur, Tarik Ainane

**Affiliations:** 1 Medicinal Research Institute, Center for Research and Study of Djibouti, BP 486, Djibouti City P.O. Box 486, Djibouti; 2 Peltier Hospital of Djibouti, Djibouti City P.O. Box 2123, Djibouti; 3 Superior School of Technology of Khenifra (EST-Khenifra), University of Sultan Moulay Slimane, BP 170, Khenifra 54000, Morocco; 4 Omics of Algae Group, Industrial Biotechnology, International Centre for Genetic Engineering and Bio-technology, Aruna Asaf Ali Marg, New Delhi 110067, India

**Keywords:** cancer, computational approach, IBProME, lamellar alkaloids, mechanism, pharmacological properties

## Abstract

This study aimed to evaluate the anticancer activity of lamellar alkaloid derivatives extracted from the tunicate *Didemnum abradatum* from Moucha Island (Djibouti), focusing on their antiviability against human cell lines and using biocomputational analyses via the Integrated Biomolecular Profiling and Mechanism Evaluation (IBProME) method to understand their mechanisms of action. Two alkaloids were isolated, lamellarin D and lamellarin T, whose structures were confirmed by state-of-the-art analytical techniques. Cell viability tests were performed on PC3, A549 and JIMT-T1 cell lines, and IBProME analyses were used to predict their interactions with p53 protein and evaluate their toxicological and pharmacokinetic profiles. The results showed that lamellarin D was particularly effective against prostate and lung cancer cells, with respective IC_50_ values of 5.25 µg/mL and 8.64 µg/mL, close to those of doxorubicin. In contrast, lamellarin T showed less marked activity but remains promising. Computational analyses via IBProME highlighted differences in chemical reactivity between the two compounds, with lamellarin D being more reactive. Toxicity tests revealed that lamellarin D exhibited lower acute toxicity than lamellarin T. In terms of pharmacokinetic properties, both molecules showed low absorption and moderate bioavailability, although lamellarin T displayed more marked lipophilicity. These results suggest that lamellars, particularly lamellarin D, have therapeutic potential for the treatment of certain types of cancer.

## 1. Introduction

Cancer is a leading cause of death worldwide, linked to uncontrolled cell proliferation [1]. This disease disrupts cell death mechanisms, allowing tumor cells to multiply [2]. Research is focused on developing treatments aimed at restoring apoptosis or targeting specific molecules such as topoisomerase I and the p53 protein [3,4].

In this perspective, marine alkaloids, and in particular lamellarins, have emerged as a promising class of bioactive compounds, thanks to their cytotoxic properties and their ability to interfere with critical cellular processes in tumor cells [5]. Lamellarins, a family of hexacyclic pyrrole alkaloids, were first isolated from marine invertebrates, such as molluscs, tunicates, and sponges, attracting considerable interest since their discovery by Faulkner in 1985 [6]. More than 50 lamellarins have been identified, belonging to the marine pyrrole alkaloid class, alongside compounds such as lukianols, polycitones, storniamides, ningalins, purpurone, and dictyodendrines [7]. These molecules, whose core is based on a pyrrolo[2,1-a]isoquinoline, exhibit significant antiviability, with IC_50_ values in the submicromolar range against various cancer cell lines [8]. Among these lamellarins, compounds D, K and M stand out for their ability to strongly inhibit topoisomerase I, a key enzyme in the management of DNA topological constraints during replication and transcription [9]. This property places them among promising candidates for the treatment of cancers resistant to conventional treatments. In addition, the development of efficient chemical synthesis routes has expanded access to a wide range of lamellarins and their analogues, thus facilitating in-depth biological studies [10].

However, although progress has been made in the characterization of lamellarins, several challenges remain that limit their clinical application. First, the selectivity of these compounds for cancer cells over healthy cells remains insufficiently documented, raising concerns regarding their safety of use [11]. Second, the exact molecular mechanisms by which these compounds exert their antineoplastic effects, including their interaction with targets such as topoisomerase I or p53 protein, still require in-depth investigation [12]. Furthermore, the variability of biological responses observed between different lamellarins and various cell lines indicates heterogeneity in their modes of action, thus complicating the optimization of their efficacy. Finally, the majority of studies conducted on lamellarins are limited to in vitro models, without sufficient data regarding their performance in preclinical or clinical settings [13].

The main objective of this research is to evaluate the antineoplastic potential of lamellar alkaloid derivatives extracted from the tunicate *Didemnum abradatum* from the sea of Moucha Island in Djibouti by analyzing their cell viability on human cancer cell lines (PC3 for prostate cancer, JIMT-T1 for breast cancer, and A549 for lung cancer) and elucidating their mechanisms of action through biocomputational analyses. The hypothesis suggests that these derivatives could possess selective antiviability towards tumor cells by interacting with important targets, such as topoisomerase I and p53 protein, which are involved in the regulation of apoptosis. To test this hypothesis, the compounds were extracted, purified, and characterized using advanced analytical techniques, such as Ultraviolet-Visible spectrophotometry (UV-VIS), Fourier transform infrared spectroscopy (FTIR), Nuclear magnetic resonance spectroscopy (NMR), and High-resolution mass spectrometry (MS). Cell viability assays were performed on the aforementioned cell lines, while biocomputational studies were conducted, including molecular docking with p53 protein and energetic analyses, as well as predictions regarding the toxicity and pharmacokinetic properties of the compounds. A multiple correlation analysis was also performed to better understand the mechanisms underlying these biological effects.

## 2. Results

### 2.1. Extraction

**Figure 1:**
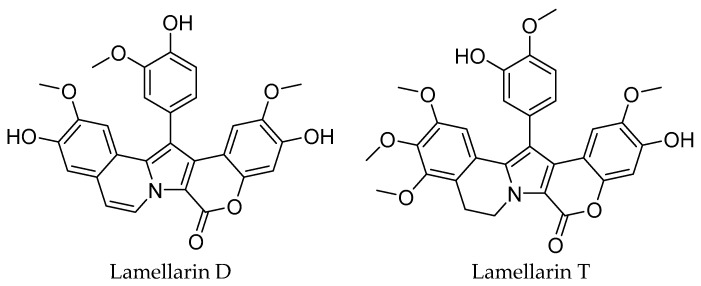
Two lamellarin alkaloid derivatives isolated from *Didemnum abradatum*.

Our study was conducted around Moucha Island, in the Gulf of Tadjourah, Djibouti, where *Didemnum abradatum* tunicates were targeted. Extraction of lamellar alkaloids was performed according to analytical protocols detailed below, resulting in the isolation of lamellarin D and lamellarin T derivatives (Figure 1), purified by high-performance liquid chromatography (HPLC), with respective yields of 0.021‰ and 0.305‰.

### 2.2. Cell Viability Assessment

In vitro tests were performed to evaluate the effect of the two extracted molecules, lamellarin D and lamellarin T, on cell viability in three human cancer lines: PC3 (prostate), A549 (lung), and JIMT-T1 (breast). These analyses were performed using the CellTiter-Glo assay, which measures intracellular ATP levels as an indicator of cell viability. This assay reflects the ability of cells to maintain their energy metabolism and is a recognized method for assessing the antiproliferative effects of compounds. However, it does not allow for direct measurement of cytotoxicity, i.e., irreversible cellular damage such as membrane lysis, typically assessed by tests such as LDH release or annexin V detection.

The results obtained (Table 1) are expressed as IC_50_, i.e., the concentration of the compound required to reduce cell viability by 50%. A lower IC_50_ reflects a more pronounced antiviability activity and allows for a comparison of the efficacy of the compounds tested.

For lamellarin D, the IC_50_ measured against PC3 cells is 5.25 ± 0.05 µg/mL, indicating a significant reduction in cell viability. This value is close to that of doxorubicin (3.10 ± 0.05 µg/mL), a reference drug in oncology, suggesting comparable efficacy in this model [14]. In contrast, against A549 cells, lamellarin D has an IC_50_ of 8.64 ± 0.10 µg/mL, indicating a moderate antiproliferative effect, but superior to that of doxorubicin (IC_50_ = 55.22 ± 0.95 µg/mL). This suggests a more marked efficacy of lamellarin D against lung cancer. Against JIMT-T1 cells, lamellarin D exhibited an IC_50_ of 40.78 ± 0.10 µg/mL, reflecting lower antiviability activity than in other models, but still higher than that of doxorubicin (89.87 ± 1.55 µg/mL).

Regarding lamellarin T, the IC_50_ against PC3 was 20.22 ± 0.45 µg/mL, approximately four times higher than that of lamellarin D, indicating weaker antiviability activity. However, this value remains lower than that of doxorubicin, suggesting some efficacy against this cell line. For A549 cells, the IC_50_ was 14.35 ± 0.35 µg/mL, reflecting moderate activity but superior to that of doxorubicin. Finally, the IC_50_ measured against JIMT-T1 was 41.17 ± 0.05 µg/mL, comparable to that of lamellarin D and still lower than that of doxorubicin. This suggests that lamellarin T may be more active than doxorubicin in this breast cancer model, with efficacy close to that of lamellarin D.

Overall, the results indicate that lamellarins, particularly lamellarin D, exhibit promising antiviability activity, with more pronounced effects than doxorubicin in lung and breast cancer models. It is important to emphasize that, although the term “cytotoxicity” is sometimes used broadly in the literature to refer to the inhibition of cell proliferation, the present study opted for more rigorous terminology to reflect the exact nature of the measurements performed.

### 2.3. Integrated Biomolecular Profiling and Mechanism Evaluation (IBProME)

#### 2.3.1. Computational Studies

**Figure 2:**
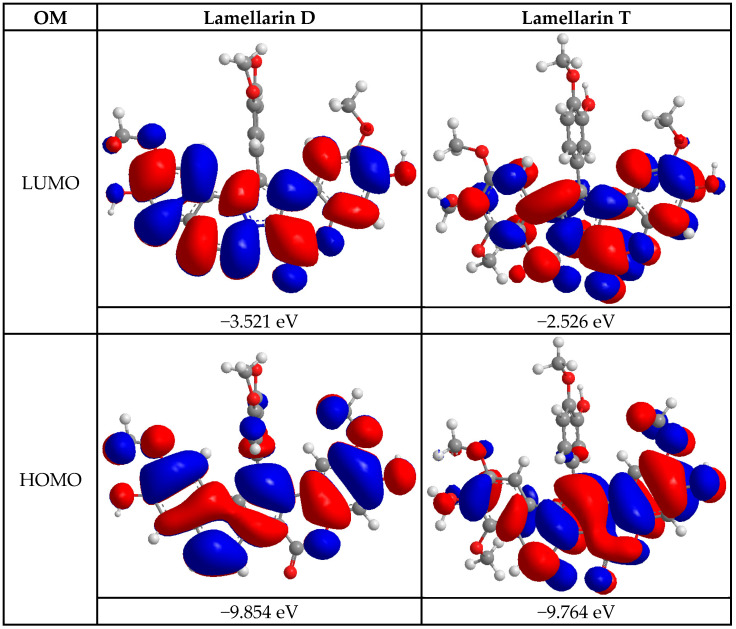
LUMO and HOMO molecular orbitals for the two compounds: lamellarin D and lamellarin T.

The first computational study consisted of the energetic analysis of the two compounds, lamellarin D and lamellarin T. Figure 2 presents the lowest (LUMO) and highest (HOMO) molecular orbital energies for these two molecules. These values were measured to assess the chemical reactivity and electronic properties of these molecules according to molecular orbital theory. Regarding the LUMO, lamellarin D exhibited a value of −3.521 eV, while lamellarin T showed a value of −2.526 eV. LUMO represents the energy of the lowest unoccupied molecular orbital, and therefore the molecule’s ability to accept electrons [15]. A higher LUMO value, such as that observed for lamellarin T, indicates a greater tendency of this molecule to accept electrons compared to lamellarin D, which has a lower LUMO. For the HOMO, lamellarin D showed a value of −9.854 eV, while lamellarin T showed a value of −9.764 eV [16]. The HOMO corresponds to the energy of the highest occupied molecular orbital, reflecting the molecule’s ability to donate electrons. A higher HOMO value, such as that of lamellarin T, suggests that this molecule may be slightly more likely to donate electrons than lamellarin D. The energy gap between the HOMO and the LUMO (HOMO-LUMO gap) was also calculated. For lamellarin D, this gap was 6.333 eV, while for lamellarin T, it was 7.290 eV. The HOMO-LUMO gap provides an indication of the molecule’s electronic stability and chemical reactivity. The larger this gap is, the more stable and less reactive the molecule. Thus, lamellarin T, with a wider band gap, could be more stable and less chemically reactive than lamellarin D, which has a smaller band gap [17]. Energy descriptors were calculated from the HOMO and LUMO values for both compounds, lamellarin D and lamellarin T (Table 2). These descriptors were used to assess the chemical reactivity and stability of these molecules. Softness (η), inversely related to chemical hardness, was measured at 3.167 eV for lamellarin D and 3.619 eV for lamellarin T. lamellarin D was therefore characterized as softer and more reactive compared to lamellarin T, which exhibited a lower softness value [18]. The Pearson reactivity index (ω) was measured to be 7.062 eV for lamellarin D and 5.217 eV for lamellarin T. This result indicated that lamellarin D exhibits a greater ability to accept or donate electrons than lamellarin T, thus suggesting a higher reactivity for lamellarin D [19]. The chemical stability descriptor (CmS) was calculated to be 0.158 eV^−1^ for lamellarin D and 0.138 eV^−1^ for lamellarin T. Although the values were close, lamellarin D showed slightly higher stability due to its larger energy gap between the HOMO and LUMO [20]. The reactivity density (RD) showed a value of 8.862 eV for lamellarin D and 13.989 eV for lamellarin T. This revealed that lamellarin D exhibits higher chemical reactivity than lamellarin T, which has a larger reactivity density, thus suggesting lower reactivity [21]. The chemical hardness (η) was measured as 3.167 eV for lamellarin D and 3.619 eV for lamellarin T. Higher chemical hardness for lamellarin T suggested higher stability and lower reactivity compared to lamellarin D [22]. Finally, the electronegativity (χ) was calculated as 6.688 eV for lamellarin D and 6.145 eV for lamellarin T. This result showed that lamellarin D exhibits a higher ability to accept electrons compared to lamellarin T. The results showed that lamellarin D is more reactive and more likely to donate or accept electrons, while lamellarin T is more stable and exhibits lower reactivity, mainly due to its higher chemical hardness and higher reactivity density [23].

#### 2.3.2. Toxicity Study and Prediction

Analysis of the acute and environmental toxicity parameters of lamellarin D and lamellarin T allowed us to assess their hazard potential, both for model organisms and the environment. The results are presented in the form of various measures, including acute toxicity in rats and environmental toxicity parameters (Table 3). Regarding acute toxicity in rats, lamellarin D showed an LD_50_ by the intraperitoneal (IP) route of 752.00 g/kg, while lamellarin T showed a much lower value of 129.40 g/kg. This result suggests that lamellarin D exhibits less acute toxicity than lamellarin T via the IP route. Regarding intravenous (IV) toxicity, lamellarin D showed an LD_50_ of 95.30 g/kg, while lamellarin T showed a value of 48.88 g/kg, which also indicates that lamellarin D is less toxic than lamellarin T via the IV route. For oral toxicity, lamellarin D showed an LD_50_ of 1521.00 g/kg, much higher than that of lamellarin T, which was 374.10 g/kg, suggesting lower oral toxicity for lamellarin D. On the other hand, for subcutaneous (SC) toxicity, lamellarin D showed an LD_50_ of 638.40 g/kg, compared to 603.70 g/kg for lamellarin T, a relatively small difference, but suggesting slightly lower toxicity for lamellarin D. For environmental toxicity parameters, the bioaccumulation factor (BCF) was measured at 3.59 for lamellarin D and 2.29 for lamellarin T. This result shows that lamellarin D has a higher bioaccumulation potential than lamellarin T, which could indicate a greater environmental risk in terms of accumulation in organisms. For toxicity to *Daphnia magna* (a model aquatic organism), lamellarin D exhibited an LC_50_ of 5.02 × 10^6^ mol/L, while lamellarin T showed a lower LC_50_ of 1.38 × 10^7^ mol/L, suggesting lower toxicity of lamellarin T to *Daphnia magna*. For toxicity to *Fathead minnow* fish, lamellarin D exhibited an LC_50_ of 8.89 × 10^−5^ mmol/L, while lamellarin T showed an LC_50_ of 4.68 × 10^−5^ mmol/L, indicating lower toxicity of lamellarin D to this model fish. Finally, for toxicity to *Tetrahymena pyriformis*, a single-celled organism used to test effects on aquatic microorganisms, lamellarin D showed an IGC_50_ of 4.04 mol/L, while lamellarin T showed a much higher IGC_50_ of 24.58 mol/L, suggesting that lamellarin D may be more toxic to this model. The results show that lamellarin D exhibits lower acute toxicity than lamellarin T in most cases, particularly via the oral and intraperitoneal routes, although both compounds show varying environmental toxicity potential. Lamellarin D may pose a greater environmental risk due to its greater bioaccumulation potential [24].

#### 2.3.3. Molecular Docking

**Figure 3:**
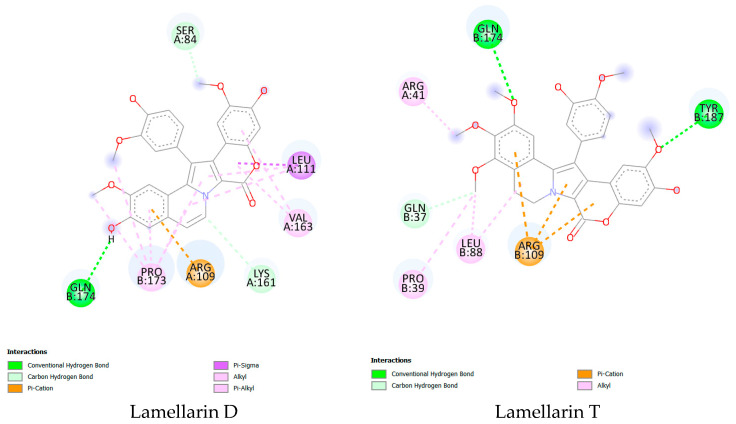
Molecular docking of lamellarin D and lamellarin T with the p53 protein. ^(*)^ A and B chains refer to distinct subunits in a multimeric protein. Each chain represents a different polypeptide sequence in the structure.

Analysis of molecular docking results as a second computational approach allowed us to examine the interactions between lamellarin D and lamellarin T with the p53 protein (Figure 3). The p53 protein is involved in cell cycle regulation and DNA repair and is frequently implicated in cancers such as HER2-positive prostate, lung, and breast cancers [25,26]. The parameters studied include binding free energy (ΔG), binding affinity (pKi), as well as the types of bonds formed between the molecules and the target protein, including hydrogen, π-cation, π-σ, alkyl, and π-alkyl bonds (Table 4). The binding free energy (ΔG) for lamellarin D was measured at −35.564 kJ/mol, while for lamellarin T, it was −33.990 kJ/mol. These negative values suggest that both molecules bind effectively to the p53 protein, with a more stable interaction observed for lamellarin D, which has a slightly more negative binding free energy [27]. However, the difference between the two values is small, indicating that both compounds exhibit strong target binding capacity. Regarding binding affinity, lamellarin D showed a pKi of 5.820, while lamellarin T showed a slightly higher value of 6.000. A higher pKi value reflects a stronger affinity, suggesting that lamellarin T may bind slightly more effectively to the p53 protein compared to lamellarin D [28]. Regarding the types of bonds formed, lamellarin T formed two conventional hydrogen bonds, while lamellarin D formed only one. This indicates that lamellarin T makes more conventional hydrogen bonds, which could stabilize its interaction with the p53 protein. In contrast, lamellarin D formed two carbon-hydrogen bonds, while lamellarin T only formed one. This difference suggests that lamellarin D may be able to make more specific interactions with the protein through these carbon-hydrogen bonds. lamellarin T also formed three π-cation bonds, while lamellarin D only formed one. π-cation bonds are important for interacting with aromatic residues in the target protein, and this difference could mean that lamellarin T interacts more effectively with aromatic residues in p53 than lamellarin D. In addition, lamellarin D formed one π-σ bond, while lamellarin T did not exhibit this type of bond. The presence of the π-σ bond for lamellarin D could influence the flexibility and deformation of the molecule during interaction with the target protein. Regarding hydrophobic interactions, lamellarin D formed five alkyl bonds, compared to four for lamellarin T. This indicates that lamellarin D may have a stronger interaction with hydrophobic groups on the surface of the p53 protein. In addition, lamellarin D formed four π-alkyl bonds, whereas lamellarin T did not form this type of bond. The π-alkyl bonds, which are important for strengthening hydrophobic interactions, suggest that lamellarin D may maintain a more stable interaction with the protein due to these additional bonds [29,30]. Although both molecules show strong affinity for the p53 protein, lamellarin T shows a slightly higher affinity, as evidenced by its higher pKi value. However, lamellarin D forms a greater number of hydrogen, alkyl, and π-alkyl bonds, which could make its interaction with the target protein more stable and stronger. lamellarin T, on the other hand, establishes more π-cation bonds, which could also strengthen the stability of its interaction with p53. These results suggest that both compounds have therapeutic potential in targeting the p53 protein for anticancer treatments.

#### 2.3.4. Pharmacokinetic Properties

Analysis of the pharmacokinetic properties of lamellarin D and lamellarin T allowed us to evaluate their behavior in the body in terms of absorption, distribution, metabolism, excretion, and physicochemical properties (Table 5). Regarding absorption, both molecules demonstrated poor gastrointestinal absorption, indicating that they may have difficulty being effectively absorbed when administered orally. Both molecules also exhibited poor water solubility, with respective Log S values of −6.78 for lamellarin D and −6.13 for lamellarin T, suggesting insufficient aqueous solubility for optimal absorption. Regarding distribution, both molecules exhibited similar lipophilicity values (Log Po/w), with lamellarin D at 4.12 and lamellarin T at 4.09, indicating that both are lipophilic and will likely distribute into fatty tissues. However, neither was able to cross the blood-brain barrier (BBB), limiting their potential in the treatment of central nervous system cancers. Furthermore, lamellarin T was identified as a substrate of P-glycoprotein (P-gp), which could lead to faster clearance of this molecule from target cells, while lamellarin D was not, which may indicate better persistence in tissues [31]. Regarding metabolism, both molecules have been identified as inhibitors of the CYP2C19 and CYP2C9 enzymes, suggesting that they may interfere with the metabolism of other drugs processed by these enzymes. Furthermore, lamellarin T inhibits CYP2D6, while lamellarin D does not. Neither compound exhibits significant inhibition of the CYP3A4 and CYP1A2 enzymes, which may reduce the risk of drug interactions with many common medications [32]. Regarding excretion, lamellarin D demonstrated a better skin permeability (Log Kp) of −5.32 cm/s compared to lamellarin T (−6.43 cm/s), suggesting that lamellarin D may be better absorbed transdermally when applied topically. The bioavailability score for both molecules was 0.55, indicating moderate bioavailability and partially limited systemic absorption. Regarding physicochemical properties, both molecules exhibited similar Topological Polar Surface Area (TPSA) values, suggesting moderate polarity properties, influencing their ability to cross biological membranes. Molar refractivity showed that lamellarin T had a slightly higher value (149.47 versus 139.43 for lamellarin D), which could indicate a larger molecular size. Regarding lipophilicity properties, lamellarin T exhibited higher values in all Log Po/w calculation methods (iLOGP, XLOGP3, WLOGP), indicating that it is slightly more lipophilic than lamellarin D, which could affect its distribution in fatty tissues and its ability to cross cell membranes [33,34]. In summary, the pharmacokinetic properties of both molecules show similar characteristics in terms of absorption, distribution, and metabolism, with notable differences in their abilities to inhibit certain enzymes and interact with biological membranes. Both molecules exhibit moderate bioavailability, but lamellarin T appears to be more lipophilic and may have a higher affinity for certain tissues, while lamellarin D exhibits better skin permeability and fewer potential drug interactions. This information is useful for evaluating the efficacy and safety of these compounds as potential anticancer treatments.

### 2.4. Multiple Correlation Study

**Figure 4:**
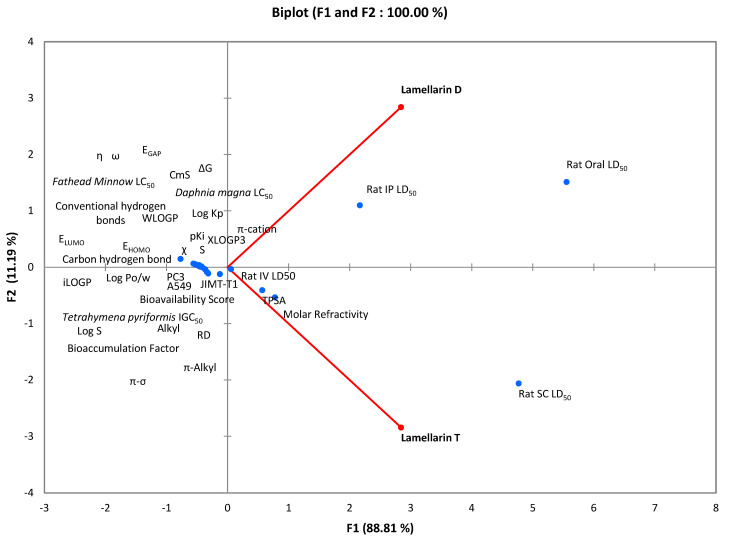
Correlation between numerical data of cytotoxic activity and biocomputational parameters of lamellarin D and lamellarin T molecules.

Multiple correlation analysis was performed through principal component analysis, which made it possible to study the relationship between cytotoxic activity and biocomputational parameters of the molecules lamellarin D and lamellarin T (Figure 4). The correlation between the data of these two compounds was evaluated at 0.776, indicating a moderate relationship between their pharmacological and toxicological characteristics. The results showed that the principal component F1 explains a large part of the variance in the data, especially for parameters such as PC3, A549, pKi, EGAP, and χ, which are highly correlated with F1. These parameters are mainly related to pharmacokinetic properties such as solubility, lipophilicity, and molecular interactions, suggesting that F1 is influenced by characteristics related to absorption, distribution, and the ability of the molecules to interact with the protein target. The F1 component could, therefore, be associated with the therapeutic efficacy of the molecules. In contrast, the F2 component showed a more marked association with toxicity properties, such as LD_50_s for different administration routes in rats, bioaccumulation factor, and LC_50_s for organisms such as *Daphnia magna* and *Fathead minnow*. These results led to the conclusion that F2 mainly represents the side effects and environmental impact of the molecules. Thus, F1 is more related to therapeutic efficacy, while F2 is more representative of the toxicity and bioaccumulation of the compounds. This information is essential to comprehensively assess the safety and efficacy of compounds in a therapeutic context, thus allowing a better understanding of their behavior in the body and anticipating their potential biological and toxic effects.

## 3. Discussion

This study evaluated the anticancer properties of lamellar alkaloid derivatives, lamellarin D and lamellarin T, extracted from the tunicate Didemnum abradatum collected from the waters of Moucha Island, Djibouti. The Integrated Biomolecular Profiling and Mechanism Evaluation (IBProME) computational method was applied to analyze their antiviability against human cell lines (PC3, A549, JIMT-T1), their mechanisms of action via interactions with p53 protein and topoisomerase I, as well as their toxicological and pharmacokinetic profiles. The initial hypothesis, which postulated selective antiviability of lamellar cells via interaction with key molecular targets involved in apoptosis and the cell cycle, was confirmed, with particularly promising results for lamellarin D.

For the first step, quantum profiling of the molecules was performed by calculating the HOMO-LUMO molecular orbital energies and the energy gap (ΔE). Lamellarin D was observed to exhibit increased chemical reactivity, attributed to a smaller HOMO-LUMO gap, facilitating interactions with biological targets such as the p53 protein. In contrast, lamellarin T exhibited superior chemical stability, which explained its less pronounced antiviability but a potential affinity for specific interactions. Molecular electrostatic potential (MEP) maps revealed electrophilic regions on lamellarin D, further supporting its potential for interaction with nucleophilic sites on target proteins [35].

Furthermore, in silico toxicity prediction allowed for the assessment of the toxicological risks of lamellarins. Lamellarin D was found to exhibit relatively low acute toxicity (high oral and intraperitoneal LD50s) compared to lamellarin T, but greater bioaccumulation, raising environmental concerns [36]. QSAR models identified potentially toxic functional groups in lamellarin T, explaining its increased risks of mutagenicity and genotoxicity. These results highlighted the need for thorough ecotoxicological assessments prior to any therapeutic application.

In the following section, biological activity prediction allowed for the prediction of the pharmacological activities of lamellarins. Lamellarin D demonstrated a high probability of anticancer activity against the PC3 (IC_50_ = 5.25 µg/mL) and A549 (IC_50_ = 8.64 µg/mL) lines, comparable to doxorubicin. These results confirmed previous data reporting nanomolar IC_50_s for lamellarin D against prostate cancer (10.9 nM) and leukemia (5 nM) lines [37]. Lamellarin T, with an IC_50_ of 20.22 µg/mL against PC3, showed lower activity, consistent with its IC_50_ of 27 µM against HeLa [38]. Probability scores allowed lamellarin D to be prioritized for therapeutic applications [39,40,41].

Furthermore, molecular docking simulations were performed to model the interactions of lamellarins with p53 and topoisomerase I. Lamellarin D showed high binding affinity with p53, characterized by hydrophobic and electrostatic interactions, thus validating its role in the induction of apoptosis and cell cycle arrest [42]. Lamellarin T showed slightly higher affinity for p53, but its interactions were less energetic, suggesting distinct mechanisms of action. Interactions with topoisomerase I confirmed the enzyme inhibition by lamellarin D, reinforcing its potential to overcome multidrug resistance (MDR) observed in lines such as CEM/C2 (IC_50_ = 0.72 µM) [43].

The results obtained via IBProME corroborated previous studies on lamellarins, recognized as highly cytotoxic marine alkaloids. Lamellarin D stands out for its efficacy against various tumor lines, including colon (IC_50_ = 9 nM) and breast (GI50 = 0.25 µM) cancer. Its ability to reverse multidrug resistance, particularly in refractory cancers, makes it a promising candidate for targeted therapies [44,45,46]. The integration of IBProME has allowed a deeper understanding of the mechanisms of action, linking the chemical reactivity of lamellarin D to its interactions with p53 and topoisomerase I, and explaining the lower efficacy of lamellarin T by its increased chemical stability.

## 4. Material and Methods

### 4.1. Analytical Apparatus Technique

In this study, several advanced techniques were used to ensure rigorous structural identification of the lamellar alkaloid derivatives. The compounds were first separated by column chromatography using Merck silica gel 60, allowing efficient separation at atmospheric pressure. Thin-layer chromatography (TLC) served as a preliminary monitoring method, ensuring constant tracking of the purification process. To refine the separation, an Agilent 1260 Infinity II HPLC system (Agilent Technologies, Santa Clara, CA, USA) was used. It allows precise fraction control thanks to a pump with a maximum pressure of 600 bar and a photodiode detector for detailed spectral analysis. The use of Luna 100 Å 10 µm semi-preparative columns resulted in the production of high-purity compounds. Spectroscopic analyses included a Shimadzu UV–1601 UV-VIS spectrophotometer (Shimadzu Corporation, Kyoto, Japan) to record characteristic absorption spectra, and FTIR analysis with a Bruker Vertex 70 spectrophotometer (Bruker Corporation, Billerica, MA, USA) to identify the functional groups of the compounds. NMR spectroscopy, performed with Bruker Avance DPX-300 and Avance III 600 spectrometers (Bruker Corporation, Billerica, MA, USA), allowed for an in-depth analysis of the chemical environments of protons and carbons. Finally, high-resolution mass spectrometry (HRMS) was performed with a Bruker BioApex 47 FT spectrometer (Bruker Corporation, Billerica, MA, USA), allowing for accurate determination of the molecular mass of the isolated compounds, thus validating their final structure.

### 4.2. Biomass

This study was conducted in the sea surrounding Moucha Island, located in the Gulf of Tadjoura, Djibouti (GPS coordinates: 11°45′06.7″ N, 43°13′51.4″ E), as part of a scientific mission planned for June 2024. The main objective was to collect *Didemnum abradatum* tunicates to explore their potential applications in the pharmaceutical field, taking advantage of the medicinal properties of local marine resources. Sampling was carried out using specialized techniques such as scuba diving and benthic dredging, covering variable bathymetric strata ranging from 0 to 30 m depth. To ensure the rigor of sampling and identification procedures, a control specimen was collected and recorded under the code 2024DA11. This specimen was transferred and preserved at the Medical Research Institute, attached to the Center for Studies and Research of Djibouti (CERD), thus ensuring the long-term preservation of data and samples in optimal conditions.

### 4.3. Extraction and Purification

The extraction of lamellarin alkaloids was carried out using a multi-phase protocol aimed at isolating and purifying secondary metabolites produced by the tunicate *Didemnum abradatum*. Biological specimens were immersed directly at the collection site in a hydroalcoholic solution composed of ethanol (Sigma-Aldrich, St. Louis, MO, USA) and water (Et-OH/H_2_O, 1:1). This mixture was concentrated on the same day by evaporation under reduced pressure (in vacuo), resulting in an aqueous suspension that was subsequently freeze-dried to obtain a dry solid residue [47].

For the extraction phase itself, 588 g (dry weight) of freeze-dried material was macerated in a binary solvent composed of methanol and chloroform (CH_3_OH/CHCl_3_, 2:1) (Sigma-Aldrich, St. Louis, MO, USA). The resulting organic phases were combined and then concentrated using a rotary evaporator under reduced pressure. A secondary extraction with absolute methanol was performed to optimize the extraction of polar compounds, producing a concentrated crude extract of 2.514 g.

This extract was fractionated by normal-phase column chromatography at atmospheric pressure, using silica as the stationary phase. Elution was performed using a solvent gradient of ethyl acetate and methanol, allowing the separation of the mixture into twelve distinct fractions, designated F1 to F12. The separation was monitored by thin-layer chromatography (TLC) (Merk, Darmstadt, Germany). Fraction F9, particularly enriched in constituents of interest, underwent an advanced purification step using high-performance liquid chromatography (HPLC) in semi-preparative mode. The system used was equipped with a high-pressure quaternary pump and a diode array detector (DAD), allowing for detailed spectral analysis. Elution was performed in two steps: an increasing methanol/water gradient, followed by an isocratic phase in a CH_3_OH/H_2_O mixture (3:1). At the end of the final purification phase, the lamellarin D (12.3 mg) and lamellarin T (179.3 mg) derivatives were isolated in chemically pure form, with respective retention times of RT_D_ = 2.3 min and RT_T_ = 5.5 min [48,49].

**Lamellarin D (7,17-dihydroxy-12-(4-hydroxy-3-methoxyphenyl)-8,16-dimethoxy-4-oxa-1-azapentacyclo [11.8.0.0^2,11^.0^5,10^.0^14,19^]henicosa-2(11),5,7,9,12,14,16,18,20-nonaen-3-one):** Yellow powder. mp > 300 °C. R_f_ (EtOAc/_,_CH_3_OH, 95:5 )= 0.62. UV (CH_3_OH) λ_max_ (nm): 385, 365, 282, 210. ^1^H NMR (DMSO δ_6_, 300 MHz) δ:3.48 (9H, s, 3×OCH_3_), 6.72 (1H, s, H), 6.87 (1H, d, *J* = 7.5 Hz, H), 6.89 (1H, dd, *J* = 7.5, 1.5 Hz, H), 7.12 (1H, d, *J* = 7.5 Hz, H), 7.19 (1H, d, *J* = 7.5 Hz, H), 7.24 (1H, d, *J* = 7.5 Hz, H), 7.43 (1H, d, *J* = 7.5 Hz, H), 8.19 (1H, d, *J* = 7.5 Hz, H), 9.48 (2H, s, OH), 9.83 (1H, s, OH). ^13^C NMR (150 MHz, DMSO-d_6_): δ 56.1 (C, 3×CH_3_), 103.8 (CH), 105.1 (CH), 105.8 (CH), 109.7 (CH), 110.8 (C, benzene), 111.7 (C), 111.9 (CH), 114.0 (CH), 115.8 (CH), 122.4 (CH), 123.0 (CH), 123.7 (C), 124.2 (C), 125.9 (C), 127.7 (C), 128.4 (C), 133.6 (C), 144.8 (C), 146.1 (C, benzene), 146.2 (C), 148.0 (C), 148.7 (C), 149.2 (C), 150.3 (C), 154.7 (C). EI-MS m/z (rel. int.): 499 (100), 466 (31), 438 (32). HRMS (EI): m/z [M^+^] found 499.1262, calcd. for C_28_H_21_NO_8_: 499.1267.

**Lamellarin T (7-hydroxy-12-(3-hydroxy-4-methoxyphenyl)-8,16,17,18-tetramethoxy-4-oxa-1-azapentacyclo [11.8.0.0^2,11^.0^5,10^.0^14,19^]henicosa-2(11),5,7,9,12,14,16,18-octaen-3-one)**: White powder. mp 216 °C. R_f_ (EtOAc/_,_CH_3_OH, 85:15 )= 0.75. UV (CH_3_OH) λ_max_ (nm): 390, 310, 274. ^1^H NMR (DMSO δ_6_, 300 MHz): δ 2.82 (d, *J* = 7.1 Hz, 2H, CH_2_), δ 3.68 (s, 3H, CH_3_), δ 3.71 (s, 3H, CH_3_), δ 3.83 (s, 6H, CH_3_), δ 3.86 (s, 3H, CH_3_), δ 4.18 (d, *J* = 7.1 Hz, 2H, CH_2_), δ 6.80 (d, *J* = 1.5 Hz, 1H), δ 6.83 (d, *J* = 7.5 Hz, 1H), δ 6.97 (d, *J* = 1.5 Hz, 1H), δ 7.09 (d, *J* = 7.5 Hz, 1H), δ 7.14 (d, *J* = 1.5 Hz, 1H), δ 7.27 (d, *J* = 1.5 Hz, 1H), δ 9.48 (s, 2H, OH). ^13^C NMR (150 MHz, DMSO-d_6_): δ 21.7 (CH_2_), δ 44.8 (CH_2_), δ 56.1 (2CH_3_), δ 60.3 (CH_3_), δ 60.8 (CH_3_), δ 113.7 (C), δ 115.3 (C), δ 120.0 (C), δ 122.3 (CH), δ 122.7 (C), δ 127.2 (C), δ 127.8 (C), δ 135.6 (C), δ 142.4 (C), δ 144.8 (C), δ 146.1 (C), δ 146.2 (C), δ 147.4 (C), δ 147.5 (C), δ 150.3 (C), δ 150.6 (CH), δ 154.7 (4×C). EI-MS m/z (rel. int.): 545 (100), 530 (35), 515 (30). HRMS (EI): m/z [M^+^] found 545.1719, calcd. for C_30_H_28_NO_9_: 545.1685.

### 4.4. Cell Viability Assay

The in vitro antiproliferative activities of lamellarin alkaloid derivatives were evaluated in three human cancer cell lines: PC3 (prostate carcinoma), JIMT-T1 (breast carcinoma), and A549 (lung carcinoma), obtained from the American Type Culture Collection (ATCC, Rockville, MD, USA). The objective of this study was to determine the ability of these derivatives to inhibit cell proliferation using a cell viability assay based on the measurement of ATP levels [50].

The cell lines were cultured in media specific to each cell type. PC3 and A549 cells were cultured in RPMI 1640 medium supplemented with 10% fetal bovine serum (FBS) (Thermo Fisher Scientific–HyClone brand, Waltham, MA, USA) and 1% glutamine (Sigma-Aldrich, St. Louis, MO, USA), while JIMT-T1 cells were cultured in DMEM medium, also supplemented with 10% FBS and 1% glutamine. No antibiotics were added to the culture media to avoid potential interference with the cell viability assay. All cultures were maintained at 37 °C in a humidified atmosphere containing 5% CO_2_. These standardized culture conditions enabled reproducible results for biomedical analyses, particularly for the assessment of cell viability.

To perform the cell viability assay, cells were seeded in 96-well plates (3 × 10^3^ cells/well) with 90 µL of culture medium. After 24 h of incubation, the cells were treated with different concentrations of lamellarin derivatives (10, 5, 1, 0.5, 0.1, 0.05, 0.01 and 0.005 µg/mL), prepared by serial dilutions in the culture medium. Controls were included in the experiment: negative controls, treated only with reagents and solvents, and positive controls, treated with doxorubicin, a well-known antiproliferative agent. After 72 h of incubation, cell viability was assessed using the CellTiter-Glo assay, which measures ATP levels as an indicator of metabolically active cells, according to the manufacturer’s instructions (Promega, Madison, WI, USA). For this purpose, 100 µL of CellTiter-Glo reagent was added, generating a luminescent signal proportional to the amount of ATP, a key marker of cell viability. Luminescence was measured using a PolarStar Omega plate reader (BMG LabTech, Ortenberg, Germany). Dose-response curves were plotted using polynomial regression of a degree greater than 2, allowing calculation of 50% inhibitory concentrations (IC_50_). Experiments were performed in triplicate to ensure accuracy and reliability of results [51,52].

### 4.5. Integrated Biomolecular Profiling and Mechanism Evaluation (IBProME)

The IBProME (Integrated Biomolecular Profiling and Mechanism Evaluation) [53] approach combines several computational tools to provide a comprehensive analysis of the biological properties and mechanisms of the action of molecules. This method integrates quantum chemistry, in silico toxicology, molecular modeling, and biological activity prediction to provide a detailed molecular profile. The IBProME approach allows for the analysis of chemical reactivity, toxicity assessment, pharmacological property prediction, and exploration of molecular interactions with biological targets, thus facilitating the discovery of new therapeutic or industrial applications.

#### 4.5.1. Quantum Profiling of Molecules

The energy analysis of the two compounds derived from lamellar alkaloids was performed using molecular orbital-based energy descriptors. The descriptors, such as the energy of the highest occupied molecular orbital (E_HOMO_) and the energy of the lowest unoccupied molecular orbital (E_LUMO_), allowed the evaluation of the electronic properties of the molecules, including their reactivity, stability, and electrochemical behavior (Table 6). These calculations were performed using a molecular mechanics model (MMFF94) within the framework of density functional theory (DFT) calculations [54].

#### 4.5.2. Toxicity Prediction

The toxicity prediction of lamellar alkaloid compounds was performed using the General Unified Structure-Activity Relationship (GUSAR 16.06) software [55]. Using QSAR/QSPR models, this software predicts the toxicity of molecules based on a large database containing information on the chemical structure of compounds and their toxicological properties. The toxicity prediction results are divided into two main categories: acute toxicity in rats and environmental toxicity. For acute toxicity in rats, predicted endpoints include the median lethal dose for intraperitoneal administration in rats (Rat IP LD_50_), the median lethal dose for intravenous administration (Rat IV LD_50_), the median lethal dose for oral administration (Rat Oral LD_50_), and the median lethal dose for subcutaneous administration (Rat SC LD_50_). For environmental toxicity, predicted endpoints include the bioaccumulation factor (BCF), which measures a chemical’s ability to accumulate in living organisms. Other endpoints include the median lethal concentration for *Daphnia magna* (DM LC_50_), an aquatic organism used for toxicity testing, and the median lethal concentration for *Fathead minnow* fish (FM LC_50_). Finally, the median inhibitory concentration for *Tetrahymena pyriformis* (TP IGC_50_), a unicellular organism used in toxicity tests, is also predicted.

#### 4.5.3. Molecular Docking Modeling

Molecular docking was performed to analyze the interactions between the ligands lamellarin D and lamellarin T and the p53 protein, specifically the T tumor cell receptor. The structure of the p53 protein, available under PDB code 6VTC, was used for this study. The objective was to explore the interactions between ligands and active sites involved in anticancer treatments. The modeling provided a better understanding of the molecular mechanisms involved in the recognition of the original neoepitope of the p53R175H mutation presented by HLA-A2 [56]. Docking calculations were performed using AutoDock 1.2.7 software, with receptor and ligand preparation using Discovery Studio. Interaction analysis was performed in 2D to better visualize the interaction points between molecules and biological targets [57].

#### 4.5.4. Pharmacokinetic Properties

The pharmacokinetic properties of the compounds were assessed using the online SwissADME tool (http://www.swissadme.ch, access to the data site from 30 January 2025 to 10 February 2025) [58]. This tool analyzed key pharmacokinetic parameters, including absorption, distribution, metabolism, and excretion (ADME), as well as physicochemical and lipophilicity parameters of the lamellar alkaloid derivatives. For absorption, the calculated parameters included gastrointestinal (GI) absorption and water solubility (Log S), providing information on the oral absorption of the compounds. Distribution was assessed through lipophilicity (Log Po/w), blood-brain barrier (BBB) permeability, and the compounds’ ability to be substrates for P-glycoprotein (P-gp). In the metabolism step, inhibition of cytochrome P450 enzymes (CYP2C19, CYP2C9, CYP1A2, CYP2D6, and CYP3A4) was assessed, as these enzymes influence the biotransformation of the compounds. Excretion was analyzed by measuring skin permeability (Log Kp). A bioavailability score was also provided, allowing for an estimation of the proportion of the administered dose reaching the active bloodstream. Finally, physicochemical properties, such as Total Polar Surface Area (TPSA) and Molar Refractivity, were considered to characterize molecular interactions and compound stability, as were several lipophilicity measures (iLOGP, XLOGP3, WLOGP), providing a better understanding of the compounds’ lipophilic behavior [59,60,61].

### 4.6. Statistical Studies

The results of all tests were subjected to statistical analysis using XLSTAT 2016 software integrated with EXCEL. Data are expressed as mean ± uncertainty, with a significance level set at 5%, based on three replicates for each experimental condition, and were processed using the Student’s t-test [62].

On the other hand, Principal Component Analysis (PCA) was used as a mathematical method to assess the correlation between the various measured parameters. This approach aims to visualize and summarize the data obtained, allowing for easier interpretation of the results. PCA thus made it possible to group and clarify information relating to the in vitro and in silico experiments, facilitating their overall analysis and interpretation. This technique helped identify underlying relationships between variables and extract key trends present in the data, thus providing a more precise overview of the phenomena studied [63].

## 5. Conclusions

This work evaluated the anticancer potential of lamellar alkaloid derivatives extracted from the tunicate *Didemnum abradatum* from the sea of Moucha Island (Djibouti) by analyzing their cytotoxic activity against human cell lines and studying their mechanisms of action through biocomputational analyses using the Integrated Biomolecular Profiling and Mechanism Evaluation (IBProME) method. The objectives of this research were successfully achieved, notably demonstrating that lamellarin D exhibits strong antiviability against prostate and lung cancer cells, comparable to that of doxorubicin, a reference chemotherapeutic agent. Although lamellarin T showed less marked activity, it also revealed promising potential, indicating that the two derivatives may have distinct or complementary mechanisms of action. Analysis of biocomputational properties showed that lamellarin D is more chemically reactive and has a greater ability to interact with key molecular targets, such as the p53 protein, involved in the regulation of apoptosis. Quantum profiling, toxicity prediction, and pharmacokinetic steps within IBProME provided a deeper understanding of the mechanisms of action of these compounds, revealing that lamellarin D is more reactive and more capable of interacting with p53, while lamellarin T exhibits greater chemical stability, thus explaining its less pronounced activities. The pharmacokinetic properties of both compounds showed low absorption and moderate bioavailability, suggesting that optimization of these parameters is necessary to improve their therapeutic efficacy. Regarding toxicity, lamellarin D demonstrated a relatively safer profile, although further studies are needed to assess its environmental impact, particularly due to its bioaccumulation potential. This study thus enriches scientific knowledge concerning marine alkaloids as potential candidates for the treatment of resistant cancers.

## Figures and Tables

**Table 1:** IC_50_ (µg/mL) values for the evaluation of lamellarin D and lamellarin T in the cell viability test against the human cancer cell lines.

Cell Line	Lamellarin D	Lamellarin T	Doxorubicine
PC3	5.25 ± 0.05	20.22 ± 0.45	3.10 ± 0.05
A549	8.64 ± 0.10	14.35 ± 0.35	55.22 ± 0.95
JIMT-T1	40.78 ± 0.10	41.17 ± 0.05	89.87 ± 1.55

**Table 2:** Energy descriptors calculated from HOMO and LUMO values for lamellarin D and lamellarin T.

Energetic Descriptors	Lamellarin D	Lamellarin T
E_HOMO_ (eV)	−9.854	−9.764
E_LUMO_ (eV)	−3.521	−2.526
E_GAP_ (eV)	6.333	7.238
η (eV)	3.167	3.619
χ (eV)	6.688	6.145
S (eV^−1^)	0.316	0.276
ω (eV)	7.062	5.217
CmS (eV^−1^)	0.158	0.138
RD (eV)	8.862	13.989

**Table 3:** Predicted Acute Toxicity and Environmental Toxicity Parameters for lamellarin D and lamellarin T.

Toxicity Type	Parameter	Lamellarin D	Lamellarin T
Acute Toxicity in Rats	Rat IP LD_50_ (g/kg)	752.00	129.40
	Rat IV LD_50_ (g/kg)	95.30	48.88
	Rat Oral LD_50_ (g/kg)	1521.00	374.10
	Rat SC LD_50_ (g/kg)	638.40	603.70
Environmental Toxicity	Bioaccumulation Factor (BCF)	3.59	2.29
	*Daphnia magna* LC_50_ (mol/L)	5.02 × 10^6^	1.38 × 10^7^
	*Fathead minnow* LC_50_ (mmol/L)	8.89 × 10^−5^	4.68 × 10^−5^
	*Tetrahymena pyriformis* IGC_50_ (mol/L)	4.04	24.58

**Table 4:** Thermodynamic parameters of molecular docking.

Parameter	Lamellarin D	Lamellarin T
ΔG (kJ/mol)	−35.564	−33.990
pKi	5.820	6.000
Conventional hydrogen bonds	1	2
Carbon hydrogen bond	2	1
π-cation	1	3
π-σ	1	0
Alkyl	5	4
π-Alkyl	4	0

**Table 5:** Comparative ADME Properties of lamellarin D and lamellarin T.

ADME Step	Parameter	Lamellarin D	Lamellarin T
Absorption	GI absorption (Gastrointestinal absorption)	Low	Low
	Log S (Water Solubility)	−6.78	−6.13
Distribution	Log Po/w (Lipophilicity)	4.12	4.09
	BBB permeant (Blood-brain barrier permeability)	No	No
	P-gp substrate (P-glycoprotein substrate)	No	Yes
Metabolism	CYP2C19 inhibitor (CYP2C19 inhibitor)	Yes	Yes
	CYP2C9 inhibitor (CYP2C9 inhibitor)	Yes	Yes
	CYP1A2 inhibitor (CYP1A2 inhibitor)	No	No
	CYP2D6 inhibitor (CYP2D6 inhibitor)	No	Yes
	CYP3A4 inhibitor (CYP3A4 inhibitor)	No	No
Excretion	Log Kp (Skin permeation)	−5.32 cm/s	−6.43 cm/s
Pharmacokinetics	Bioavailability Score	0.55	0.55
Physicochemical Properties	TPSA (Topological Polar Surface Area)	123.00 Å^2^	121.75 Å^2^
	Molar Refractivity	139.43	149.47
Lipophilicity Properties	Log Po/w (iLOGP)	3.73	4.19
	Log Po/w (XLOGP3)	5.67	4.51
	Log Po/w (WLOGP)	5.16	5.09

**Table 6:** Energy descriptors.

Descriptor	Symbol	Formula
Energy gap	E_GAP_	$E_{LUMO}-E_{HOMO}$
Chemical hardness	η	$\frac{E_{LUMO}-E_{HOMO}}{2}$
Electronegativity	χ	$-\left(\frac{E_{LUMO}+E_{HOMO}}{2}\right)$
Softness	S	$S=\frac{1}{\eta }$
Pearson Reactivity Index	ω	$\omega =\frac{\chi^{2}}{2\eta }$
Chemical Stability	CmS	$CmS\sim \frac{1}{E_{GAP}}$
Reactivity Density	RD	$RD=\frac{E_{HOMO}}{E_{LUMO}}\times \eta$

## Data Availability

Data are contained within the article.
